# Association of the *WNT3* Variations and the Risk of Non-Syndromic Cleft Lip and Palate in a Population of Iranian Infants

**Published:** 2018

**Authors:** Homa Farrokhi Karibozorg, Nahid Masoudian, Kioomars Saliminejad, Asghar Ebadifar, Koorosh Kamali, Hamid Reza Khorram Khorshid

**Affiliations:** 1. Department of Biochemistry, Islamic Azad University, Damghan Branch, Damghan, Iran; 2. Reproductive Biotechnology Research Center, Avicenna Research Institute, ACECR, Tehran, Iran; 3. Dentofacial Deformities Research Center, Research Institute of Dental Sciences, Shahid Beheshti University of Medical Sciences, Tehran, Iran; 4. Genetic Research Center, University of Social Welfare and Rehabilitation Sciences, Tehran, Iran

**Keywords:** Genome wide association study, Cleft lip/Palate, Polymorphism, *WNT3*

## Abstract

**Background:**

Nonsyndromic cleft lip and/or palate (NSCL/P) is the most common orofacial birth defect, often attributed to ethnic and environmental differences. Up to now, linkage analyses and genome-wide association studies have identified several genomic susceptibility regions for NSCL/P. The *WNT* genes including *WNT3* are strong candidates for NSCL/P, since they are involved in regulating mid-face development and upper lip fusion. This study tested association of the *WNT3* polymorphisms, rs-3809857 G/T and rs9890413 G/A, with the risk of NSCL/P in a population of Iranian infants.

**Methods:**

The allelic and genotypic frequencies for each participant were determined in 113 unrelated Iranian subjects with NSCL/P and 220 control subjects using PCR and restriction fragment length polymorphism (RFLP) methods. A p-value of ≤0.05 was considered statistically significant.

**Results:**

The *WNT3* rs3809857 GT genotype was significantly lower (p=0.039, OR=0.55, 95% CI=0.30–0.97) in the NSCL/P (21.2%) than the control group (30.42%). For the *WNT3* rs9890413 G/A polymorphism, neither genotype nor allele frequencies were significantly different between the case and control groups.

**Conclusion:**

Our results indicated that the *WNT3* rs3809857 GT genotype may have a protective effect against NSCL/P in Iranian population.

## Introduction

Clefts of the lip and/or palate (CL/P) are one of the most common birth defects which are typically classified in syndromic and more common non-syndromic (NSCL/P) forms 1. NSCL/P is a complex malformation caused by the interaction of multiple genes and environmental factors 2; however, its etiology still remains poorly characterized 1. Genetic factors are thought to contribute to the development of this disorder, because the risk of recurrence of CL/P within a family is higher than for the general population 3,4.

Up to now, several distinct genetic and environmental risk factors have been identified and confirmed for the NSCL/P 1,5,6. Many candidate genes and loci have also been associated with facial clefts, through various genetic approaches. Until now, over 40 genes, including interferon regulatory 6 (*IRF6*), Msh homeobox homolog 1 (*MSX1*), transforming growth factor alpha (*TGFa*), and domain containing 2 (*CRISPLD2*) as well as the 8q24 and 17q22 loci have been suggested to be associated with the etiology of NSCL/P 1.

The *WNT* gene family plays an important role in murine craniofacial embryogenesis 7. Canonical WNT signaling is activated during midfacial morphogenesis in mice 8. Although not implicated by Genome-Wide Association (GWA) studies, Single Nucleotide Polymorphism (SNP) within the *WNT* genes has been reported to be associated with NSCL/P, and the haplotype of the *WNT* genes may explain the etiology of NSCL/P 9. SNPs in *WNT3A*, *WNT5A*, and *WNT11* are significantly associated with NSCL/P 10. Previous studies have shown that two SNPs in the *WNT3A* gene*,* rs752107 and rs3121310, were significantly associated with NSCL/P in a Chinese population 11. In another study in a northeast Chinese population C392T SNP in the *WNT10A* gene was associated with NSCL/P 12.

According to our knowledge, a few studies have evaluated the association between the *WNT3* gene and NSCL/P in Caucasian populations. The *WNT3* rs3809857 was highly associated with NSCL/P in a Polish population 9. The *WNT3* rs142167 and rs9890413 were shown to be associated with NSCL/P in a population of Caucasian ancestry 13.

The aim of this study was to evaluate the association between the two SNPs of *WNT3*, rs3809857 and rs-9890413, with the risk of NSCL/P using PCR and Restriction Fragment Length Polymorphism (RFLP) in an Iranian population.

## Materials and Methods

### Subjects

This case-control study tested the association of the *WNT3* SNPs with the risk of NSCL/P in a population of Iranian infants. The allelic and genotypic frequencies were determined in 113 unrelated newborns with NSCL/P and 220 newborns as control subjects without clefting.

A clinical examination was performed to look for dysmorphic features such as lip pits. Cases were excluded from the study if there was evidence of other facial or skeletal malformations (such as lip pits, congenital heart lesion, *etc*), metabolic or neurologic disorders or anomalies of other organ systems. Samples were recruited from Mofid Hospital, a referral pediatric center in Tehran, Iran during 2013–2015. Ethical approval for the study was obtained from the Ethics Committee of the School of Dentistry, Shahid Beheshti University of Medical Sciences. Informed consent was obtained from all parents.

### DNA extraction and genotyping

Four to ten *ml* of peripheral blood samples were collected in tubes containing 200 *μl* of 0.5 *M* EDTA and genomic DNA was extracted from peripheral blood using the salting out method as described previously 14. DNA concentration was determined by measuring the absorbance at 260 *nm*. DNA purity was measured by calculating the ratio of absorbance at 260/280 *nm*.

Genotyping of the *WNT3* rs3809857 G/T and rs-9890413 G/A SNPs in the *WNT3* was performed by PCR-RFLP. PCR-RFLP allows rapid detection of point mutations based on unique patterns of restriction enzyme cutting in specific regions of DNA. After the amplification of genomic sequences by PCR, the mutation was discriminated by digestion with specific restriction endonucleases and was identified by gel electrophoresis. This method is especially useful in studies of complex genetic diseases 15.

Briefly, a total volume of 20 *μl* containing 30 *ng* of genomic DNA, 10 *pmol* of each primer, 0.5 *μl* dNTPs mix, 2 *μl* of 10×buffer and 1 *U* of Taq DNA polymerase with 1.5 *mM* MgCl_2_ and sterile distilled water up to 20 *μl* were mixed for amplification of the target sequences (All reagents were from Bioneer, South Korea). Amplification conditions started with an initial denaturation step of 4 *min* at 94°*C*, followed by 30 cycles of denaturation at 94°*C* (45 *s*), annealing of 61°*C* (40 *s*) and 72°*C* extension (30 *s*), ended by a final 72°*C* extension for 5 *min*. The PCR products of the rs-3809857 G/T and rs9890413 G/A SNPs were digested with the BamHI and MspI restriction enzymes at 37°*C* overnight (New England BioLabs, Beverly, MA, USA). The PCR products were subjected to 8% polyacrylamide gel electrophoresis and stained with silver nitrate. The primer sequences and the pattern of restriction fragments are shown in table 1.

**Table 1.** Primer sequences and their related PCR product sizes for the *WNT3* rs3809857 and rs9890413 polymorphisms

**Variants**	**Primer sequences (5′→3′)**	**Size (*bp*)**	**Restriction enzymes**	**RFLP fragments (*bp*)**
**rs3809857**	GGTCATCGTCTCTGCATGTG CTTCCTTCTTGCAGCACTCG3	285	BamHI	GG:285
				GA: 150+135+285
				AA: 150+135
**rs9890413**	CTCTCTTCCTGCCCCAGTC AGGACAGGGCTAGGGAGTG	177	MspI	GG:78+99
				GA: 177+78+99
				AA: 177

### Statistical Analysis

A p-value of ≤0.05 was considered statistically significant. The Chi square (*χ*^2^) and Fisher’s exact tests using the SPSS statistical software (Version 11.0, Chicago, IL, USA) were performed to compare genotype and allele frequencies in the study groups. The sample was stratified by gender, and genotype and allele frequencies were compared between the study groups.

## Results

Descriptive analysis showed that among all 113 NSCL/P infants, 54 (47.8%) and 53 (46.9%) were boys and girls, respectively, while among them six had unknown gender (5.3%). Of all 220 healthy controls, 217 had known gender: 111 males (50.5%) and 106 females (48.2%), while three (1.3%) had unknown gender.

The distributions of genotype using chi-square showed that in the control group for the *WNT3* rs-9890413 G/A was in Hardy-Weinberg equilibrium (p-value= 0.115).

The allelic and genotypic frequencies of the *WNT3* SNPs are shown in table 2. The *WNT3* rs3809857 GT genotype was significantly lower (P=0.039, OR=0.55, 95% CI=0.30–0.97) in the NSCL/P (21.2%) than the control group (30.42%). For the *WNT3* rs9890413 G/A SNP, neither genotype nor allele frequencies were significantly different between the case and control groups (Table 3).

**Table 2.** Genotype distribution and allele frequency of the *WNT3* rs3809857 polymorphism in the case and control groups

**Genotype/allele groups**	**Cases (n=113)**	**Controls (n=217)**	**p-value**	**OR (95% CI)**
**GG**	60 (53.1%)	91 (41.9%)	Reference genotype^*^	
**GT**	24 (21.2%)	66 (30.4%)	**0.039**	0.55 (0.30–0.97)
**TT**	29 (25.7%)	60 (27.7%)	0.269	0.73 (0.42–1.30)
**G**	144 (63.7%)	248 (57.1%)	Reference allele^**^	
**T**	82 (36.3%)	186 (42.9%)	0.103	0.76 (0.55– 1.06)

* The reference genotype has the highest frequency in the genotypes group.

** The reference allele has the higher frequency between the two alleles.

**Table 3.** Genotype distribution and allele frequency of the rs9890413 polymorphism in the case and control groups

**Genotype/allele groups**	**Cases (n=113)**	**Controls (n=220)**	**p-value**	**OR (95% CI)**
**AA**	71 (62.8%)	138 (62.7%)	Reference genotype ^*^	
**AG**	34 (30.1%)	71 (32.3%)	0.778	0.9 (0.56–1.53)
**GG**	8 (7.1%)	11 (5%)	0.476	1.4 (0.54– 3.60)
**A**	178 (78.8%)	347 (78.9%)	Reference allele ^**^	
**G**	48 (21.2%)	93 (21.1%)	0.976	1.01 (0.70–1.50)

* The reference genotype has the highest frequency in the genotypes group.

** The reference allele has the higher frequency between the two alleles.

When the samples were stratified by gender, the *WNT3* rs3809857 GT genotype in the male sub-group was significantly lower than the one in the case and the control group (p-value=0.037, OR=0.42, 95% CI=0.20–0.96). When stratified by gender, the *WNT3* rs9890413 did not differ significantly between cases and controls in the male and female sub-groups.

## Discussion

NSCL/P is a genetically complex disorder caused by the interaction of multiple genetic and environmental factors 16. Among non-syndromic clefts, cleft lip/palate is twice more frequent in males than in females, while the cleft lip is twice as frequent in females 17. The genes contributing to the etiology of NSCL/P have been investigated by linkage analysis, genomic rearrangements, candidate genes analysis and genome-wide association studies 18. So far, several candidate genes including *MSX1*, *FGFR1*, *FGF8*, *BMP4* as well as *WNT* gene family have successfully been associated with the CL/P 10,19.

In the present study, the two *WNT3* gene polymorphisms (rs3809857 and rs9890413) were investigated to determine if any of them are implicated in the etiology of NSCL/P. Our results showed that the *WNT3* rs3809857 was associated with NSCL/P. The *WNT3* rs-3809857 GT genotype was significantly lower (p= 0.039, OR=0.55, 95% CI=0.30–0.97) in the NSCL/P than the control group. This result means that the individuals with the GT genotype showed approximately a two-fold decreased risk of NSCL/P. However, no association between the *WNT3* rs9890413 G/A polymorphism and NSCL/P was found. When stratified by gender, the *WNT3* rs9890413 did not differ significantly between cases and controls in the male and female sub-groups. When stratified by gender, the *WNT3* rs3809857 GT genotype in the male sub-group was significantly lower in the case than the control group (p-value= 0.037, OR=0.42, 95% CI=0.20–0.96).

There are a few studies which evaluated the association of *WNT* genes with the risk of NSCL/P. Our results are consistent with the results of the studies conducted by Mostowska *et al* 9 and Lu *et al* 20. This indicated that the common G allele may be a risk factor, and the minor T allele may be a protective factor for NSCL/P 20. The minor allele frequency (MAF) of *WNT3* rs9890413 in *WNT3* northeast China (MAF= 0.03) is lower than the one in the European American (MAF=0.363), and Caucasian populations (MAF=0.32). This distinction may be attributed to the population heterogeneity between the northeast China and the western countries 20.

The *WNT* gene family plays a critical role in the development of the lip and ectoderm. The *WNT* gene family consists of structurally related conserved genes which encode secreted signaling glycoproteins proteins that play a fundamental role in developmental and cell-biological processes 21,22. The binding of Wnt ligands to Frizzled receptors, a family of G protein-coupled receptor, activates several distinct intracellular signaling pathways, such as the best understood canonical Wnt/b-catenin pathway 23. Liu and Millar 24 reported that the dynamic activation of the WNT/beta-catenin signaling pathway was correlated with the occurrence of a cleft lip and cleft palate. Abnormal Wnt signaling has been associated with many human diseases, such as cancer, degenerative diseases, or osteoporosis 22,25,26. Wnt signaling also plays a crucial role in various aspects of craniofacial development, which was suggested by studies of phenotypes observed in knockout mouse embryos 23,26. Mutations and markers in genes encoding Wnt pathway 27 components might be correlated with oral congenital anomalies, including cleft lip and palate 24. Cleft lip with cleft palate may be induced by genetic inactivation of Lrp6, a co-receptor of the *WNT*/beta-catenin signaling pathway 28.

NSCL/P risk factors differ among populations which confirm the importance of testing putative susceptibility variants in different genetic backgrounds 29. Epidemiological data support a role for the environmental risk factors in the development of orofacial clefts. Maternal smoking has been consistently associated with an increased risk of clefting, with a population-attributable risk estimated as high as 20% and an odd ratio of 1.3 for the CL/P 30. Nutrition during pregnancy has been suggested as another contributing factor based on observational and interventional studies using folate supplements as a preventive measure 31. The beneficial effect of folate use, however, remains controversial and has not been consistently replicated 18.

## Conclusion

Mutations and polymorphisms in components of Wnt signaling in NCL/P are not strong. Genome-wide association studies for CL/P did not confirm any role of the WNT signaling genes in the etiology of NSCL/P. Accordingly, further studies are required to explore the role of *WNT* genes during human craniofacial development and to identify possible functional variants and/or haplotypes in these genes that may influence the risk of NSCL/P in different populations 9.

In conclusion, the *WNT3* rs3809857 SNP was associated with the non-syndromic CL/P and the *WNT3* GT genotype may have a protective effect against NSCL/P in Iranian population.
